# Constructing “Closed” and “Open” {Mn_8_} Clusters

**DOI:** 10.1021/acs.cgd.2c00489

**Published:** 2022-07-05

**Authors:** Thomais G. Tziotzi, Athanasios Mavromagoulos, Mark Murrie, Scott J. Dalgarno, Marco Evangelisti, Euan K. Brechin, Constantinos J. Milios

**Affiliations:** † Department of Chemistry, The University of Crete, Voutes, Herakleion 71003, Greece; ‡ School of Chemistry, University of Glasgow, University Avenue, Glasgow G12 8QQ, Scotland, U.K.; § Institute of Chemical Sciences, Heriot-Watt University, Riccarton, Edinburgh, EH14 4AS, Scotland, U.K.; ∥ Instituto de Nanociencia y Materiales de Aragón, CSIC − Universidad de Zaragoza, Zaragoza 50009, Spain; ⊥ EaStCHEM School of Chemistry, The University of Edinburgh, David Brewster Road, Edinburgh, EH9 3FJ, Scotland, U.K.

## Abstract

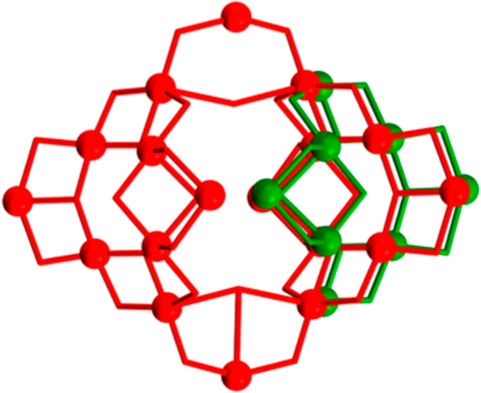

Use of the 1,3,5-tri(2-hydroxyethyl)-1,3,5-triazacyclohexane ligand, LH_3_, in manganese chemistry affords access to two structurally related {Mn_8_} clusters: a “closed” {Mn^III^_6_Mn^II^_2_} puckered square wheel of formula [Mn_8_L_2_(LH)O_3_(OH)_2_(MeO)_2_Br(imH)(H_2_O)_3_](Br)_3_ (**1**; imH = imidazole) and an “open” {Mn^III^_8_} rod of formula [Mn^ΙΙΙ^_8_L_2_O_4_(aibH)_2_(aib)_2_(MeO)_6_(MeOH)_2_](NO_3_)_2_ (**2**, aibH = 2-amino-isobutyric acid). In each case the triaza ligands, L/LH, direct the formation of {Mn_3_} triangles with their N atoms preferentially bonding to the Jahn–Teller axes of the Mn^III^ ions. Subsequent self-assembly is dependent on the anion of the Mn salt and the identity of the organic coligand employed—the terminally bonded imidazole and the chelating/bridging amino acid. The {Mn_3_} triangles fold up on themselves in **1**, forming a wheel. However, the syn, syn-bridging carboxylates in **2** prevent this from happening, instead directing the formation of a linear rod. Magnetic susceptibility and magnetization measurements reveal competing ferro- and antiferromagnetic interactions in both complexes, the exchange being somewhat weaker in **1** due to the presence of Mn^II^ ions.

## Abstract 2

Employment of the 1,3,5-tri(2-hydroxyethyl)-1,3,5-triazacyclohexane ligand in manganese chemistry leads to the synthesis of two related {Mn_8_} complexes; a “closed” {Mn^III^_6_Mn^II^_2_} puckered square wheel and an “open” {Mn^III^_8_} rod. In both clusters the ligand assembles oxo-centered triangular [Mn^III^_3_O(L)]^4+^ units in which the triaza N atoms favor coordination on the Jahn−Teller axes of the Mn^III^ ions leading to coparallel alignment of the JT axes.

## Introduction

**Scheme 1:**
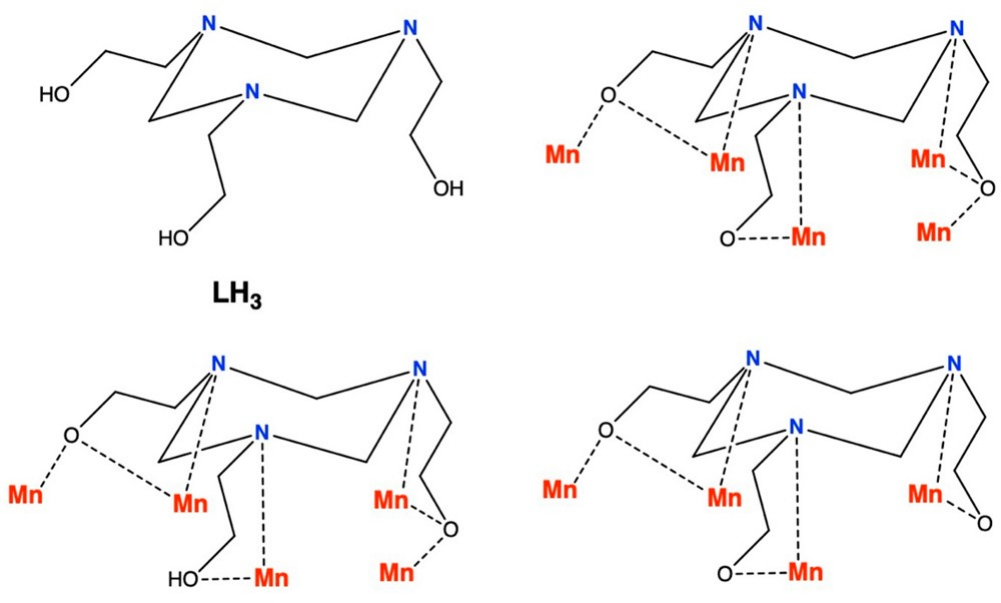
Ligand 1,3,5-Tri(2-hydroxyethyl)-1,3,5-triazacyclohexane, LH_3_, and Its Coordination Modes Found in **1** and **2**

The chemistry of polymetallic manganese compounds constitutes a vibrant and growing area of research that has characterized species of nuclearities up to 84.^1^ These aesthetically pleasing structures represent a breadth of metallic topologies constructed from both mono- and multimetallic building blocks.^2−4^ Those of low nuclearity have been proposed as mimics for the active site of Photosystem II in which a pentanuclear [Mn_4_Ca] complex is responsible for the oxidative splitting of water to molecular oxygen, protons, and electrons, upon solar irradiation.^5−14^ Manganese clusters of all nuclearities have also been at the heart of molecular magnetism, having provided the prototype single-molecule magnet, [Mn_12_], which gave rise to an exciting research area that persists to this day.^15−18^ Given that the topology of a polymetallic cluster depends on the identity and oxidation state of the metal ion, the presence/absence of oxide/hydroxide ions, the coordination ability/directionality of the organic/inorganic ligands used, and subtle changes in the reaction conditions employed, exploring the coordination chemistry of newly designed ligands alongside physical characterization of the products made remains a key initial step. This remains fundamentally important for the development of magneto-structural correlations, which play a pivotal role in understanding the structural factors underpinning magnetic behavior, a prerequisite for any potential application.^19^ Following this approach, we report the synthesis of two related octanuclear Mn clusters upon employment of the ethanolamine-containing ligand 1,3,5-tri(2-hydroxyethyl)-1,3,5-triazacyclohexane ligand, LH_3_, (Scheme 1), as a means of further investigating and expanding the chemistry and coordination ability of this ligand.^20−22^

## Experimental Section

All synthetic procedures were performed under aerobic conditions using materials and solvents as received. LH_3_ was prepared as previously reported.^23^

### [Mn_8_L_2_(LH)O_3_(OH)_2_(MeO)_2_Br(imH)(H_2_O)_3_](Br)_3_ (1)

MnBr_2_·4H_2_O (0.5 mmol, 143 mg), LH_3_ (0.5 mmol, 109 mg), NEt_3_ (1.5 mmol), and imidazole (imH, 0.5 mmol, 34 mg) were stirred in MeCN/MeOH (1:1, 20 mL), forming a pale olive-brown solution that was left stirring for 45 min at room temperature. The resulting dark brown solution was then filtered and left undisturbed to evaporate slowly at room temperature. Dark brown single crystals suitable for X-ray crystallography were formed after 4 d in 30–35% yield. Anal. Calcd for C_32_H_71_Br_4_Mn_8_N_11_O_19_ (**1**): C, 22.97; H, 4.28; N, 9.21%. Found: C, 23.09; H, 4.17; N 9.03%.

### [Mn^ΙΙΙ^_8_L_2_O_4_(aibH)_2_(aib)_2_(MeO)_6_(MeOH)_2_](NO_3_)_2_ (2)

Mn(NO_3_)_2_·6H_2_O (0.5 mmol, 143 mg), LH_3_ (0.5 mmol, 109 mg), 2-amino-isobutyric acid (aibH, 0.25 mmol, 25.7 mg), and NEt_3_ (1.5 mmol) were added in MeOH (20 mL), and the resulting dark brown solution was left to stir for 45 min. The solution was then filtered and left undisturbed to evaporate at room temperature. Dark brown single crystals suitable for X-ray crystallography were formed after 5 d in 35–40% yield. Anal. Calcd for C_42_H_96_Mn_8_N_12_O_32_ (**2**): C, 29.32; H, 5.62; N, 9.77%. Found: C, 29.20; H, 5.51; N 9.86%.

### Physical Methods

Elemental analyses (C, H, N) were performed by the University of Ioannina microanalysis service. Variable-temperature, solid-state direct current (dc) magnetic susceptibility data were collected on a Quantum Design MPMS-XL magnetometer at the University of Zaragoza and a Quantum Design MPMS3 magnetometer at the University of Glasgow. Diamagnetic corrections were applied to the observed paramagnetic susceptibilities using Pascal’s constants. Powder X-ray diffraction (PXRD) measurements were collected on freshly prepared samples of the complexes on a PANanalytical X’Pert Pro MPD diffractometer at the University of Crete.

### Single-Crystal X-ray Diffraction

Diffraction data for **1** and **2** were collected on a Bruker D8 Venture diffractometer (University of Crete), equipped with a PHOTION II CPAD detector at 210 and 200 K, respectively. Hydrogen atoms were modeled in idealized geometries, except those of waters of crystallization, which were located in the Fourier maps. Data collection parameters and structure solution and refinement details are presented in Table S1, while full crystallographic details may be found in the Supporting Information (CIF files with CCDC reference numbers 2159430 and 2159431 correspond to **1** and **2**, respectively).

## Results and Discussion

### Synthesis

The 1:1 reaction of MnBr_2_·4H_2_O with LH_3_ in a basic MeOH/MeCN solution, and in the presence of imidazole, imH, leads to the formation of black crystals of [Mn^III^_6_Mn^II^_2_L_2_(LH)O_3_(OH)_2_(MeO)_2_Br(imH)(H_2_O)_3_](Br)_3_ (**1**) after 4 d in good yield. Complex **1** is a mixed-valent octanuclear complex. Base-assisted aerial oxidation of the Mn(II) ions occurs readily, even in the presence of the mildly reducing Br^–^ ions. Interestingly, attempts to change the identity of the product and the Mn^III^/Mn^II^ ratio by changing reaction conditions and reactant stoichiometry failed. Indeed compound **1** was isolated in all cases, as evidenced by a PXRD comparison between **1** and the crystalline materials obtained, perhaps highlighting the stability of the structure. Analogous reactions under solvothermal conditions led to amorphous (pale brown) powders and/or the dissociation of the LH_3_ ligand to its component parts.^24^ This appears to be a common theme in the chemistry of LH_3_, and it may be attributed to the reducing environment created by the high-pressure/temperature conditions in the autoclave. With the identity and structure of **1** established (vide infra) the next step was to attempt to replace the terminally bonded imidazole ligand with a bridging ligand, in this case 2-amino-isobutyric acid, to examine how this would affect the self-assembly process. Thus, the reaction between Mn(NO_3_)_2_·6H_2_O, LH_3_, and aibH in a basic MeOH solution affords the octanuclear complex [Mn^ΙΙΙ^_8_L_2_O_4_(aibH)_2_(aib)_2_(MeO)_6_(MeOH)_2_](NO_3_)_2_ (**2**). The Mn ions in **2** are all in the 3+ oxidation state, and the triaza ligands are now fully deprotonated, with the cluster adopting a different topology to that seen in **1**, in which analogous building blocks have self-assembled in a different manner. As with complex **1**, changing reaction conditions and reactant stoichiometry did not lead to the isolation of any other crystalline species.

### Description of Structures

**Figure 1:**
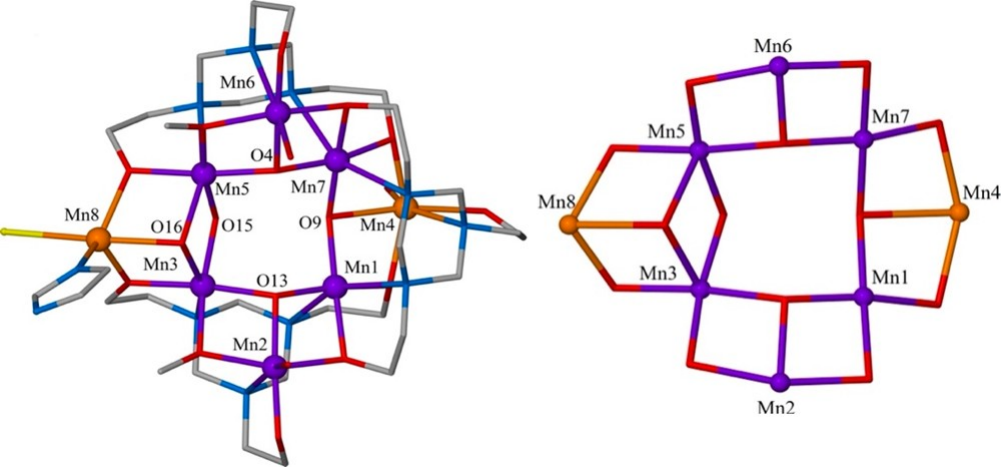
Molecular structure of the trication of **1** (left) and its metal–oxygen core (right). Color code: Mn^III^ = purple, Mn^II^ = orange, O = red, N = blue, C = gray, Br = yellow. H atoms are omitted for clarity.

Complex **1** crystallizes in the orthorhombic space group *P*2_1_2_1_2_1_. The metallic skeleton (Figure 1) of the cluster describes a wheel of vertex-sharing {Mn_3_} triangles or, alternatively, a “closed”, puckered, square [Mn^III^_6_Mn^II^_2_] wheel, with the two divalent Mn ions (Mn8 and Mn4) located on opposite sides of the square. Its metal–oxygen core consists of a twisted, asymmetric {Mn^III^_6_Mn^II^_2_(μ_3_-O)_3_(μ_3_-OH)(μ-ΟΗ)(μ-OMe)_2_(μ-O_R_)_6_}^6+^ unit in which the Mn ions forming the square [Mn^III^_6_Mn^II^_2_] core are bridged by a combination of oxide, hydroxide, and alkoxide groups. More specifically, in the upper and lower corners of the square wheel, the two groups of three Mn^III^ centers (Mn1, Mn2, Mn3 and Mn5, Mn6, Mn7) are bridged by one μ_3_-O^2–^ (O4 and O13, respectively), one μ-OMe (O5 and O14, respectively), and one μ-O_R_ (O6 and O8, respectively). In the left corner of the square wheel, consisting of Mn5, Mn3, and Mn8, the Mn centers are bridged by one μ_3_-OH^–^ (O16), one μ-OH^–^ (O15), and two μ-O_R_ groups. The right corner of the square wheel, consisting of Mn1, Mn4, and Mn7, is held together by one μ_3_-O^2–^ (O9) and two μ-O_R_ (O1, O12) bridges. The ligand is found in its fully deprotonated L^3–^ and partially deprotonated LH^2–^ forms, adopting the η^2^:η^2^:η^1^:η^1^:η^1^:η^1^:μ_5_ coordination mode in both cases. The assignment of the Mn oxidation states was performed on the basis of bond valence sum (BVS) calculations (Table S2) and the appearance of Jahn–Teller (JT) axes for the six-coordinate trivalent metal ions. Αll the Mn ions are six-coordinate, with the exception of Mn8, which is five-coordinate, adopting a trigonal bipyramidal geometry. The coordination sphere of Mn8 is completed by the presence of one terminal Br^–^ anion and one terminal imH ligand. The structure of **1** displays two very interesting features: (i) the N atoms of the L^3–^/LH^2–^ ligands prefer bonding to the JT positions of the Mn^III^ ions, creating triangular building blocks, as has been previously reported,^20−22^ which self-assemble via vertex sharing, and (ii) the metallic skeleton of **1** describes half the metallic skeleton of the recently reported complex [Mn^III^_12_Mn^II^_6_(O)_6_(OH)_2_(OMe)_6_(L)_4_(LH)_2_Br_12_]^22^ (Figure S1) likely due to the presence of the terminal imH/Br^–^ ligands on Mn8 blocking the dimerization.

The terminal bonded H_2_O ligand and the monodentate O arm of the LH ligand on Mn6 are H-bonded to the equivalent atoms on Mn2 on neighboring clusters (O(H)···O, ∼2.6 Å), with the former also being H-bonded to the Br counteranions (O(H)···Br, ∼2.6 Å). The result is the formation of H-bonded zigzag chains of clusters running along the *b*-axis of the cell in the extended structure (Figure S2).

**Figure 2:**
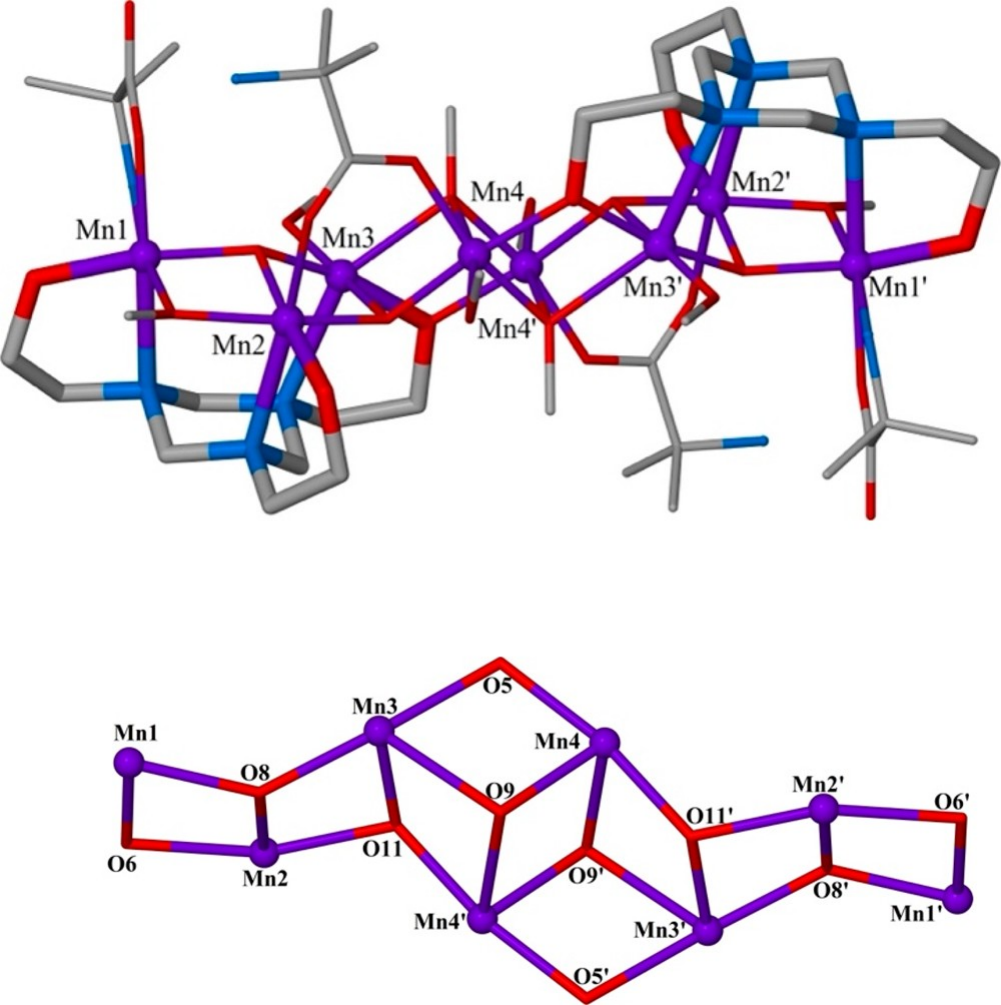
Molecular structure of the dication of **2** highlighting the capping mode of the L^3–^ ligand (top) and its metallic skeleton (bottom). Color code: Mn^III^ = purple, Mn^II^ = orange, O = red, N = blue, C = gray. H atoms are omitted for clarity.

**Figure 3:**
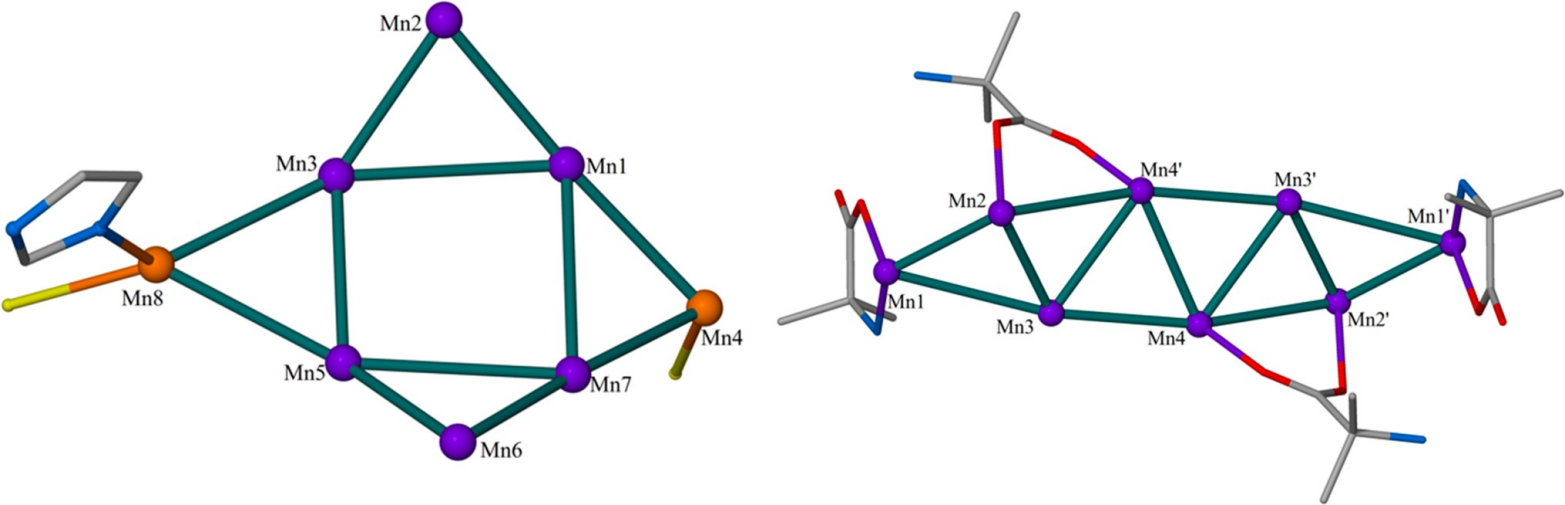
A comparison of the “wrapped” vs “linear” octametallic cores of **1** (left) and **2** (right).

Complex **2** crystallizes in the triclinic space group *P*1̅. Its metallic skeleton (Figure 2) describes six edge-sharing triangles arranged in an “open” or rod-like fashion and possessing a {Mn^III^_8_(μ_3_-O)_4_(μ_3_-OMe)_2_(μ-O_R_)_2_(μ-OMe)_2_} core. The ligand is found in its fully deprotonated form, L^3–^, adopting an η^2^:η^1^:η^1^:η^1^:η^1^:η^1^:μ_4_ bonding mode, “capping” a metallic {Mn_3_} triangle via the three N atoms and two monodentate arms, and further bridging to a central Mn ion through the remaining arm. Two of the amino acid ligands are found in the zwitterionic form, aibH, adopting an η^1^:η^1^:μ coordination mode, while the remaining two ligands are in the monoanionic form, aib^–^, and bonding in chelate fashion forming a five-membered ring via the amino group and one O_carboxylate_ atom. All the Mn ions are in the 3+ oxidation state (Table S2), six-coordinate, and in distorted octahedral geometries, with their JT axes being approximately coparallel, lying perpendicular to the mean plane of the {Mn_8_} rod. As in **1**, the N atoms of the L^3–^ ligand display a preference for occupying solely the JT positions on the Mn^III^ ions, creating [Mn_3_] triangular building blocks. However, on this occasion they self-assemble in a linear fashion rather than “wrapping up” to form a wheel, as illustrated in Figure 3. Given the similarity of the reactions, this suggests the process is governed to a large extent by the nature and bonding preferences of the different organic coligands employed. For example, the presence of the syn, syn-bridging carboxylates in **2** may favor the assembly of a more linear cluster through a promotion of edge-sharing rather than vertex-sharing triangles, preventing cyclization (Figure 3).

The NH_3_^+^ moiety of the zwitterionic, bridging aibH ligand is H-bonded to the terminally bonded O arm of the chelating aib ligand in the same molecule (N(H)···O, ∼2.9 Å). They are also H-bonded to the MeOH molecules of crystallization (N(H)···O, ∼2.9 Å) and to the non-coordinating carboxylate O atom of an aibH ligand on a neighboring molecule (N(H)···O, ∼2.8 Å). The latter results in the formation of staggered chains of clusters in the extended structure of **2** (Figure S3).

### Magnetic Properties

**Figure 4:**
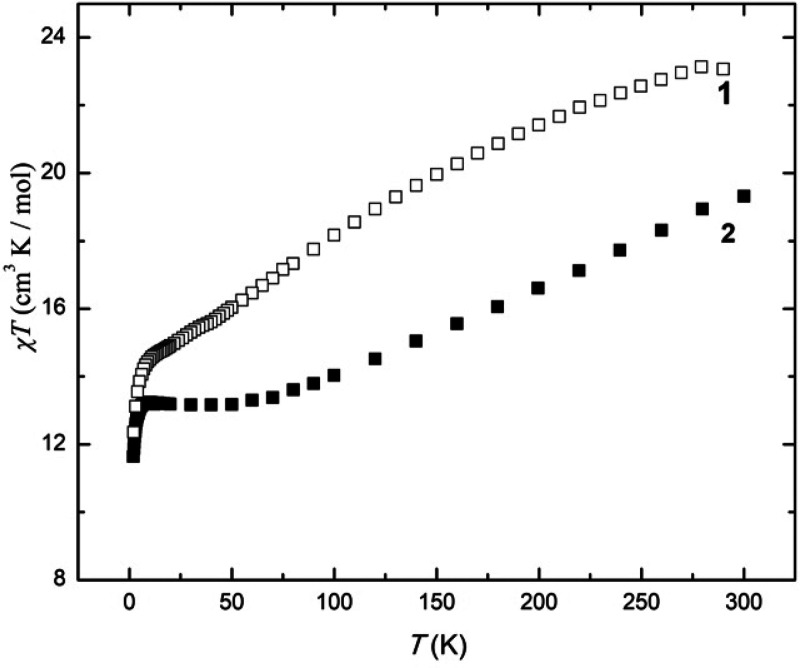
Temperature dependence of the *χT* product, where χ is the dc molar magnetic susceptibility, for **1** and **2**, as labeled, collected in an applied magnetic field of *B* = 0.1 T.

Variable-temperature dc magnetic susceptibility data were collected for microcrystalline samples of **1** and **2** in the temperature range 2–300 K under an applied magnetic field of 0.1 T and are plotted as the *χT* product versus *T* in Figure 4. The purity of the samples were confirmed by means of PXRD comparison with the simulated data from the single-crystal structures (Figure S4). For both complexes, the room-temperature values of *χT* (**1**, 23.08 cm^3^ K mol^–1^, **2**: 19.31 cm^3^ K mol^–1^) are slightly lower than the theoretical values expected for non-interacting [Mn^III^_6_Mn^II^_2_] (26.75 cm^3^ K mol^–1^) and [Mn^III^_8_] (24.00 cm^3^ K mol^–1^) units, respectively, assuming *g* = 2.00. Both complexes show similar variable-temperature behavior: when cooled, *χT* decreases steadily before it plateaus between 50 and 10 K for **1** at 14.5 cm^3^ mol^–1^ K and between 100 and 10 K for **2** at ∼13.4 cm^3^ K mol^–1^. Below 10 K, *χT* decreases rapidly to values of ∼12.4 cm^3^ K mol^–1^ (**1**) and ∼11.6 (**2**) cm^3^ K mol^–1^. This behavior suggests the dominance of relatively strong antiferromagnetic exchange interactions in both **1** and **2** with the plateaus attributed to competing ferro- and antiferromagnetic interactions within the clusters. The exchange appears to be weaker in **1** than **2**, which is likely due to the presence of the Mn^II^ ions, which are known to mediate rather weak nearest-neighbor exchange coupling.^25,26^

**Figure 5:**
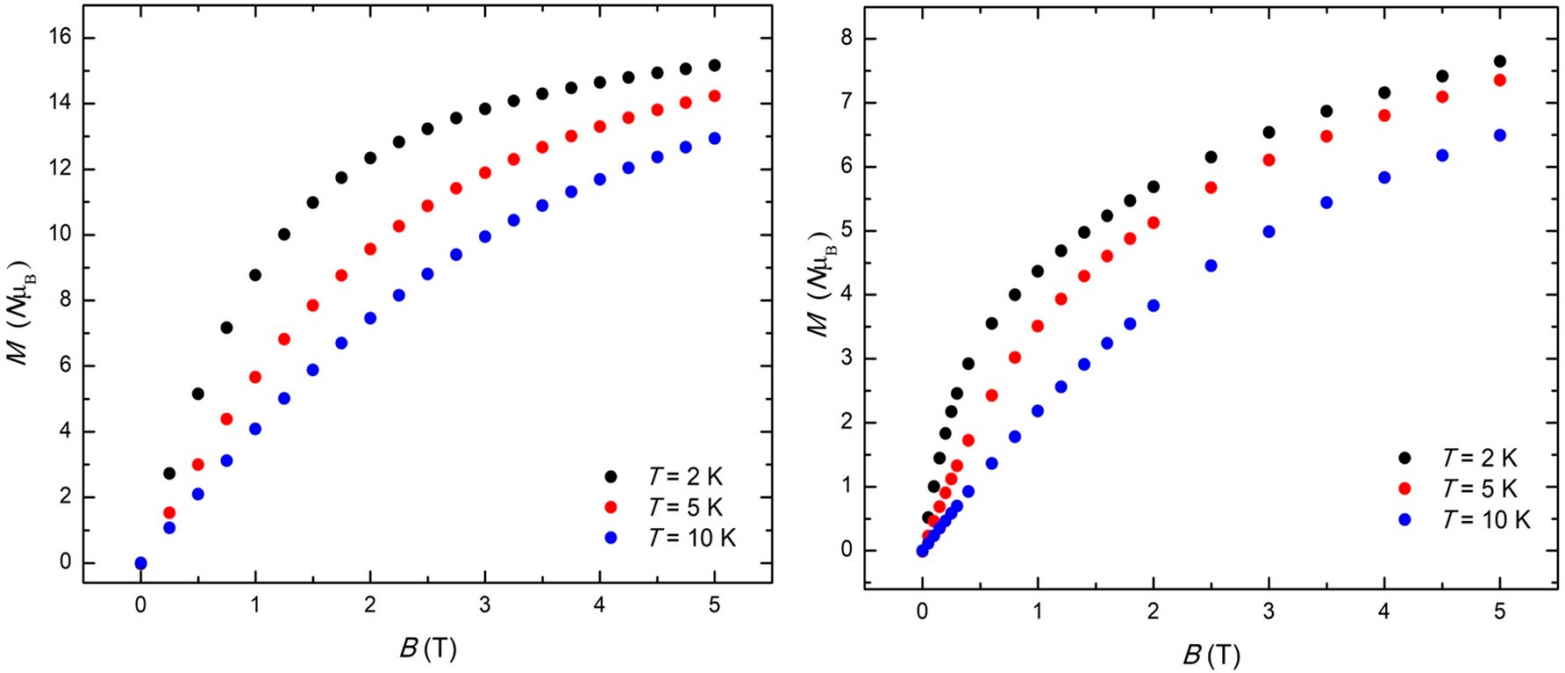
Isothermal molar magnetization *M* vs applied magnetic field data for **1** (left) and **2** (right), collected for *T* = 2, 5, and 10 K, as labeled.

Low-temperature, variable-temperature, and variable-field magnetization data were measured for both clusters in the temperature range 2–10 K and in magnetic fields up to 5 T (Figure 5). At the lowest temperature and highest field measured, *M* reaches values of ∼15.2 and ∼7.7 μ_B_ for **1** and **2**, respectively. This is indicative of the presence of dominant antiferromagnetic exchange and relatively small spin ground states, in agreement with the susceptibility data and previous magneto-structural correlations of alkoxide-bridged [Mn^III^_2_] dimers with parallel JT axes oriented perpendicular to the bridging plane (Type I dimers)^27,28^ and related complexes.^29,30^ The large nuclearity and complicated topology/structure of the two compounds precludes any quantitative analysis of the data. No out-of-phase ac susceptibility signals were detected under zero-applied dc fields for either complex.

## Conclusions

Replacing imH with aibH in the reaction between a Mn^II^ salt and LH_3_ leads to the formation of a linear [Mn_8_] “rod” rather than a [Mn_8_] “wheel”. The building blocks in each case are {Mn^III^_3_} triangles directed by the L/LH ligands, which preferentially bond to the JT axes of the Mn^III^ ions. The subsequent self-assembly process is then dictated by the anion of the Mn salt (Br^–^ vs NO_3_^–^) and the organic coligands employed, with the latter clearly having a huge influence on topology. While the Br^–^ and imH ligands are monodentate, allowing the {Mn_3_} triangles to self-assemble via vertex sharing into a wheel, the syn, syn-bridging aib and chelating aibH ligands direct the formation of a linear or rod-like structure containing edge-sharing {Mn_3_} triangles. The presence of the aibH coligand also leads to an increased oxidation state level in **2** ([Mn^III^_8_]) versus **1** ([Mn^III^_6_Mn^II^_2_]) and an increased oxide content. The magnetic behavior of the two complexes is, perhaps unsurprisingly, rather similar but with the Mn^II^ ions in **1** leading to a weaker antiferromagnetic exchange than that present in **2**. The structural similarity of **1** and **2** with previously published structures of LH_3_^20−22^ highlights the dominant topological role played by the ligand, which makes understanding and exploiting self-assembly processes somewhat simpler. In turn, this should allow for the synthesis of more targeted species.

## Supporting Information Available

BVS calculations for complexes **1** and **2**, data collection parameters and structure solution and refinement details for complexes **1** and **2**, overlay of the metallic cores displayed by complex **1** and the recently reported complex [Mn^III^_12_Mn^II^_6_(O)_6_(OH)_2_(OMe)_6_(L)_4_(LH)_2_Br_12_],^22^ packing diagrams for **1** and **2**, and powder XRD patterns for **1** and **2** (PDF)
