# Supplementary material for: Constructing “Closed” and “Open” {Mn8} Clusters
Source: Cryst Growth Des. 2022 Jul 5;22(8):4935–40. doi: 10.1021/acs.cgd.2c00489 (PMC9374326; doi:10.1021/acs.cgd.2c00489)
Supplement: Supplementary file 1 — cg2c00489_si_001.pdf [file cg2c00489_si_001.pdf]

Supporting Information for

Constructing “closed” and “open” {Mn_8} clusters

Thomais G. Tziotzi,[‡] Athanasios Mavromagoulos,[†] Mark Murrie, [†] Scott J. Dalgarno, [§] Marco Evangelisti,[&] Euan K. Brechin[#] and Constantinos J. Milios*[‡]*

[‡]Department of Chemistry, The University of Crete, Voutes, 71003, Herakleion, Greece. E-mail: komil@uoc.gr

[†]School of Chemistry, University of Glasgow, University Avenue, Glasgow, G12 8QQ, Scotland, UK.

[§]Institute of Chemical Sciences, Heriot-Watt University, Riccarton, Edinburgh, EH14 4AS, Scotland, UK.

[&]Instituto de Nanociencia y Materiales de Aragón (INMA), CSIC – Universidad de Zaragoza, 50009 Zaragoza, Spain.

[#]EaStCHEM School of Chemistry, The University of Edinburgh, David Brewster Road, Edinburgh, EH9 3FJ, Scotland, UK. E-mail: ebrechin@ed.ac.uk

Table S1. Crystallographic data for complexes **1-2**.

	**1**	**2**
Empirical formula[a]	$C_{32}H_{73.5}Br_4Mn_8N_{11}O_{21.75}$	$C_{42}H_{96}Mn_8N_{12}O_{32}$
Formula weight	1719.67	1720.82
Temperature/K	210(2)	200(2)
Crystal system	Orthorhombic	Triclinic
Space group	$P2_12_12_1$	P-1
a/Å	15.4108(3)	12.2551(9)
b/Å	17.6870(4)	13.9333(10)
c/Å	22.8088(5)	14.0367(11)
α/°	90	90.865(2)
β/°	90	109.313(2)
γ/°	90	106.038(2)
Volume/Å^3	6217.0(2)	2158.9(3)
Z	4	1
ρ_{calc} g/cm^3	1.837	1.324
μ/mm^{-1}	16.435	1.203
F(000)	3426	888
Crystal size/mm^3	0.33 × 0.3 × 0.21	0.28 × 0.22 × 0.18
Radiation	CuKα (λ = 1.54178)	MoKα (λ = 0.71073)
2Θ range for data collection/°	6.324 to 136.582	4.604 to 56.634
Index ranges	-18 ≤ h ≤ 18, -21 ≤ k ≤ 20, -27 ≤ l ≤ 27	-16 ≤ h ≤ 16, -18 ≤ k ≤ 18, -18 ≤ l ≤ 18
Reflections collected	47504	59890
Independent reflections	10911 [R_{int} = 0.0499, R_{sigma} = 0.0506]	10728 [R_{int} = 0.0334, R_{sigma} = 0.0264]
Data/restraints/parameters	10911/41/735	10728/0/431
Goodness-of-fit on F^2	1.042	1.028
Final R indexes [I>=2σ (I)]	R_1 = 0.0410, wR_2 = 0.1139	R_1 = 0.0413, wR_2 = 0.1093
Final R indexes [all data]	R_1 = 0.0424, wR_2 = 0.1151	R_1 = 0.0504, wR_2 = 0.1152
Largest diff. peak/hole / e Å^{-3}	1.11/-0.89	1.24/-1.25

Table S2. BVS calculations for **1** and **2**

Complex 1

	**II**	**III**	**IV**
Mn1	3.09	2.85	2.80
Mn2	3.31	3.05	2.99
Mn3	3.32	3.06	3.00
Mn4	1.95	1.80	1.76
Mn5	3.33	3.07	3.01
Mn6	3.32	3.06	3.01
Mn7	3.19	2.94	2.88
Mn8	1.77	1.56	1.54
O4	1.98		
O13	2.01		
O16	0.97		
O9	1.73		

Complex 2

	**II**	**III**	**IV**
Mn1	3.27	3.02	2.96
Mn2	3.41	3.14	3.09
Mn3	3.32	3.06	3.00
Mn4	3.37	3.10	3.05
O11	1.91		
O9	2.03		

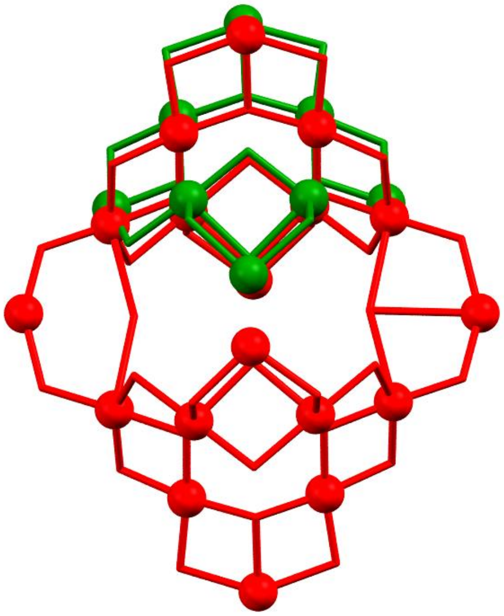

Figure S1. Overlay of the metallic cores displayed by complex **1** and the recently reported complex $[Mn^{III}_{12}Mn^{II}_{6}(O)_6(OH)_2(OMe)_6(L)_4(LH)_2Br_{12}]$,[1] showing that the metallic core of **1** describes half the metallic skeleton of the octadecanuclear complex. Colour code: skeleton of **1** = green, skeleton of the $\{Mn_{18}\}$ cluster = red.

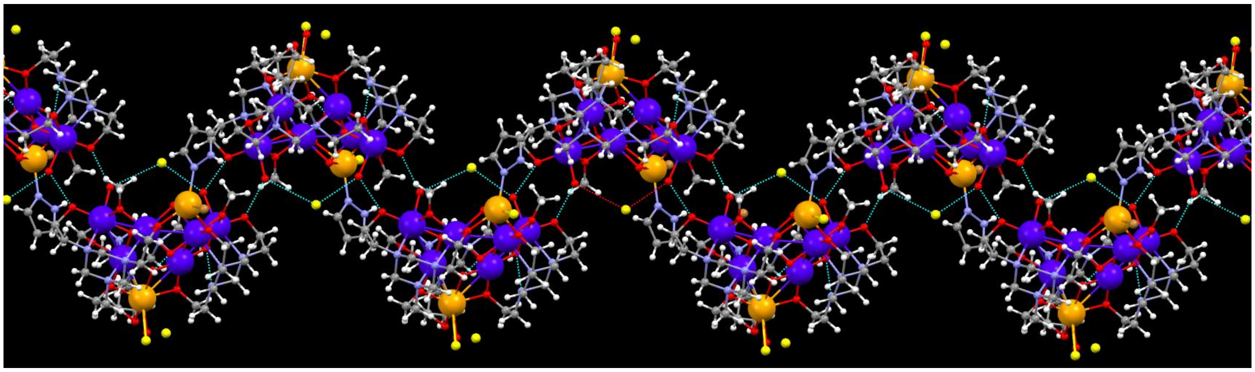

Figure S2. H-bonded zig-zag chains along the *b*-axis of the cell in the extended structure of **1**. Color code: Color code: Mn^{III} = purple, Mn^{II} = orange, O = red, N = blue, C = gray, Br = yellow, H= white.

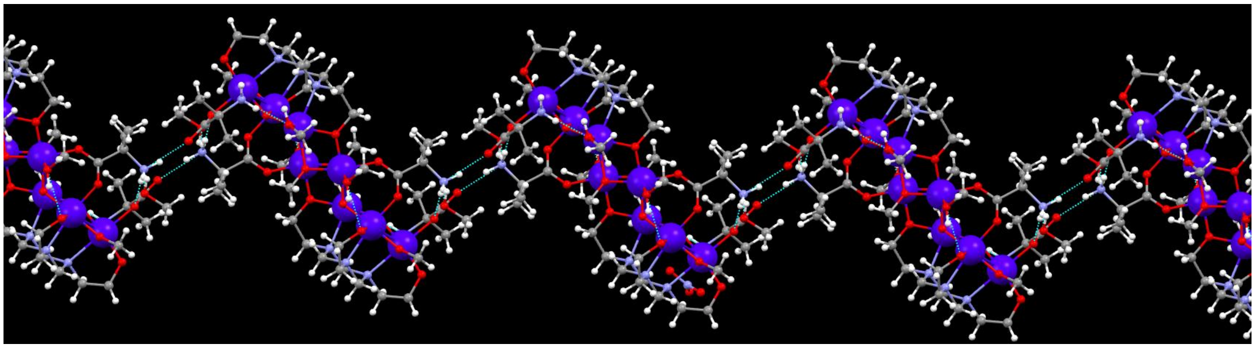

Figure S3. Staggered chains of clusters in the extended structure of **2**. Color code: Color code: Mn^{III} = purple, O = red, N = blue, C = gray, H= white.

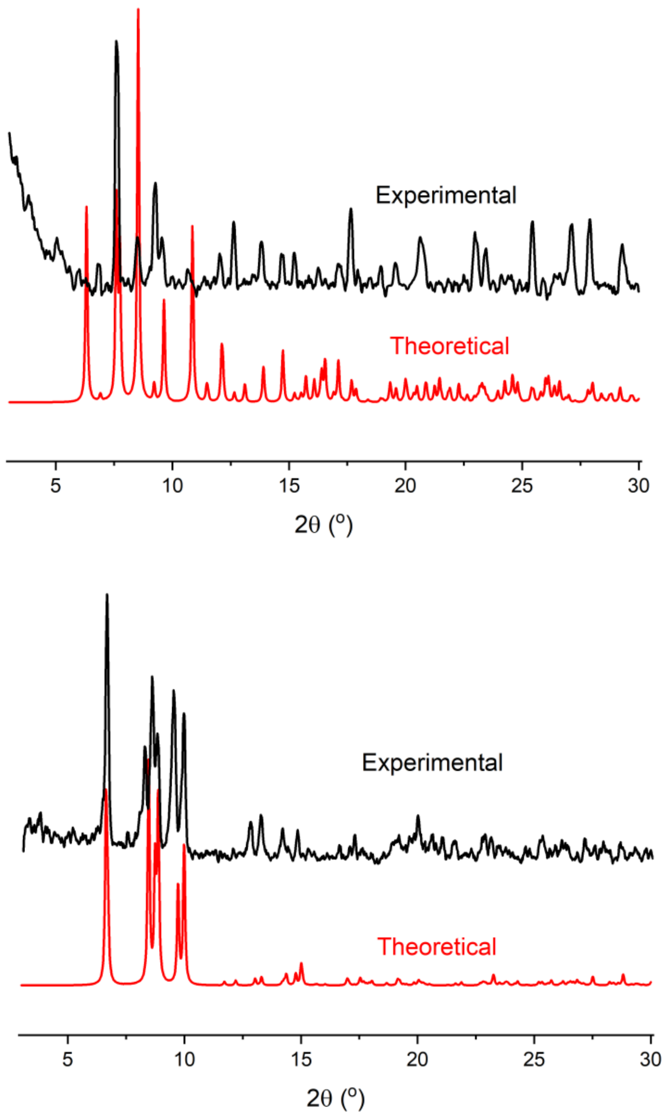

Figure S4. Powder XRD patterns for **1** (top) and **2** (bottom) and their simulated pXRD diagram. The differences in the peaks' intensity are due to solvent loss and preferred crystal orientation.

References

(1) Coletta, M.; Tziotzi, T. G.; Gray, M.; Nichol, G. S.; Singh, M. K.; Milios, C. J.; Brechin, E. K. A [Mn_{18}] wheel-of-wheels. *Chem. Commun.* 2021, **57**, 4122-4125.
